# Substance Abuse in Teenage Pregnancies – The Current Role of Psycho-Education

**DOI:** 10.1192/bjo.2025.10232

**Published:** 2025-06-20

**Authors:** Natasha Singh, Prutha Desai, Alexandra Minseo Kim, Shabbir Amanullah

**Affiliations:** 1 Dartford and Gravesham NHS Trust, Dartford, United Kingdom; 2 East London NHS Foundation Trust, London, United Kingdom; 3 University of Central Lancashire, Preston, United Kingdom; 4 Woodstock General Hospital, Ontario, Canada

## Abstract

**Aims:** To explore the current availability of psycho-education as a primary prevention against substance misuse in unplanned pregnancies for adolescents in the UK. It was noted that 80% of young adults registered with drugs and alcohol services started misusing substances under the age of 15 years. This poses an apparent risk of young mothers who are abusing drugs to have unplanned pregnancies. Moreover, pregnancies occurring sooner than desired are associated with higher risks to the health of the fetus because of delayed recognition. A study from the United States shows that women who reported using hard drugs, cannabis or smoking cigarettes at age 18 had an increased likelihood of risk behaviours and subsequent unplanned pregnancies.

**Methods:** The National Office of Statistics and the UK Government website were scanned to retrieve data appropriate to adolescent substance misuse and unplanned pregnancies. It was found that the conception rate for women aged under 18 years in England and Wales in 2021 was 13.2 per 1,000 women. Additionally, 11,326 young people were found to be in contact with alcohol and drug services between April 2021 and March 2022. Out of which 234 (5%) were young mothers or pregnant.

**Results:** The need for psychological intervention for young women focussing on substance misuse has been an ongoing dilemma. The UK government introduced programmes such as the 10-year Teenage Pregnancy Strategy for England which aimed to reduce the conception rate for women under 18 years. However, medical services often do not have data on substance misuse in young people with unplanned pregnancies as they have not had prior contact with the service. This puts them at increased risk of health complications during the pregnancy and ultimately risk of neonatal withdrawal syndromes. This demonstrates an increasing requirement to introduce psycho-education accessible to adolescents between 16–18 years to create awareness of risks of substance misuse and unplanned pregnancies.

**Conclusion:** Even though there are programmes preventing and educating regarding unplanned pregnancies, there is little support available to adolescent mothers who have ongoing substance misuse behaviours. Misusing substances while pregnant poses a risk not just to the fetus, but increases the complexities of bearing the responsibility of being a parent with a misuse disorder. Therefore, there is an emergent need for psycho-education accessible to adolescents to educate them against substance misuse during unplanned pregnancies.

